# Gene expression meta-analysis reveals aging and cellular senescence signatures in scleroderma-associated interstitial lung disease

**DOI:** 10.3389/fimmu.2024.1326922

**Published:** 2024-01-25

**Authors:** Monica M. Yang, Seoyeon Lee, Jessica Neely, Monique Hinchcliff, Paul J. Wolters, Marina Sirota

**Affiliations:** ^1^Division of Rheumatology, Department of Medicine, University of California, San Francisco, San Francisco, CA, United States; ^2^Division of Pulmonary, Critical Care, Allergy and Sleep Medicine, Department of Medicine, University of California, San Francisco, San Francisco, CA, United States; ^3^Division of Pediatric Rheumatology, Department of Pediatrics, University of California, San Francisco, San Francisco, CA, United States; ^4^Division of Rheumatology, Allergy and Immunology, Department of Internal Medicine, Yale School of Medicine, New Haven, CT, United States; ^5^Department of Pediatrics, University of California, San Francisco, San Francisco, CA, United States; ^6^Bakar Computational Health Sciences Institute, University of California, San Francisco, San Francisco, CA, United States

**Keywords:** systemic sclerosis, interstitial lung disease, aging, cellular senescence, gene expression

## Abstract

Aging and cellular senescence are increasingly recognized as key contributors to pulmonary fibrosis. However, our understanding in the context of scleroderma-associated interstitial lung disease (SSc-ILD) is limited. To investigate, we leveraged previously established lung aging- and cell-specific senescence signatures to determine their presence and potential relevance to SSc-ILD. We performed a gene expression meta-analysis of lung tissues from 38 SSc-ILD and 18 healthy controls and found that markers (GDF15, COMP, and CDKN2A) and pathways (p53) of senescence were significantly increased in SSc-ILD. When probing the established aging and cellular senescence signatures, we found that epithelial and fibroblast senescence signatures had a 3.6- and 3.7-fold enrichment, respectively, in the lung tissue of SSc-ILD and that lung aging genes (*CDKN2A*, *FRZB*, *PDE1A*, and *NAPI12)* were increased in SSc-ILD. These signatures were also enriched in SSc skin and associated with degree of skin involvement (limited vs. diffuse cutaneous). To further support these findings, we examined telomere length (TL), a surrogate for aging, in the lung tissue and found that, independent of age, SSc-ILD had significantly shorter telomeres than controls in type II alveolar cells in the lung. TL in SSc-ILD was comparable to idiopathic pulmonary fibrosis, a disease of known aberrant aging. Taken together, this study provides novel insight into the possible mechanistic effects of accelerated aging and aberrant cellular senescence in SSc-ILD pathogenesis.

## 1 Introduction

Systemic sclerosis (SSc) is an autoimmune disease characterized by diffuse fibrosis and vasculopathy that has the potential to affect nearly every organ system. Interstitial lung disease (ILD) is present in up to 90% of SSc patients with many presenting early in their disease course with variable trajectories (1–3). Despite advances in the field, ILD remains the leading cause of SSc morbidity and mortality accounting for up to 35% of SSc-related deaths and a 5-year mortality rate ranging between 18% and 44% (4, 5). The management of SSc-ILD remains challenging due to significant disease heterogeneity and lack of precision medicine approaches. An improved understanding of the molecular mechanisms underpinning SSc-ILD is needed to advance treatment in the field.

There is a growing interest in the role of aging and cellular senescence in the development and progression of pathological lung remodeling pulmonary fibrosis. Multiple epidemiological studies have shown age as a risk factor for lung fibrosis and portends a worse prognosis in lung disease (6, 7). Aged lungs have been shown to have shortened telomeres and increased expression of the cellular senescence markers p16 and p21 (8, 9). Idiopathic pulmonary fibrosis (IPF), the most common form of ILD, is characterized by telomere dysfunction and senescence programming and is a disease of older age (10–12). A defining feature in IPF is that alveolar type II cells exhibit telomere shortening, adopt features of senescent cells, and lose their ability to proliferate (13, 14). These processes are increasingly implicated across several forms of ILD, including connective tissue disease ILDs (15, 16). Better understanding of these shared mechanisms and how they may relate to SSc-ILD has prognostic and treatment implications for an otherwise rare and understudied disease.

Transcriptomic studies have examined gene expression changes during lung aging and senescence, establishing signatures that can be used to probe existing datasets. Utilizing bulk RNAseq of healthy lungs across the lifespan, Lee et al. showed that both cellular senescence and profibrotic pathways are increased with age and identified a lung-specific aging signature (8). The 22-gene signature was validated in the Gtex lung dataset with a subset of genes also found to be present in aging skin. DePianto et al. generated *in vitro* senescent cells to define an epithelial senescence, fibroblast senescence, and consensus senescence signature, which was recapitulated in a scRNAseq data from IPF and, to a lesser degree, SSc-ILD (17). While these mechanisms have been examined in murine models and in other human fibrotic lung diseases, their relevance to SSc-ILD has yet to be established.

Studies of lung tissues in SSc-ILD are limited due to disease rarity and tissue scarcity. Meta-analysis of publicly available data provides a practical approach for understanding gene expression changes in SSc-ILD and performing cross-tissue comparisons, such as the skin and blood. To date, gene expression meta-analyses in SSc-ILD have taken a largely exploratory approach, primarily focused on gene expression changes at large and shared features between tissues (18, 19). Aging and senescence signatures have yet to be probed in the existing published data. Therefore, applying a hypothesis driven approach by investigating gene signatures relevant to pulmonary fibrosis in both *in vitro* and *in vivo* studies, additional insight can be obtained into the pathogenesis of SSc-ILD and other SSc organ involvement.

In this study, we performed a gene expression meta-analysis of lung tissue from SSc-ILD to examine if aging and senescence-related mechanisms are present in the SSc-ILD compared to healthy controls. We leveraged established lung-specific aging and cellular senescence signatures that have yet to be examined in the existing SSc-ILD data. To validate our findings, we measured telomere length in SSc-ILD, IPF, and healthy control lungs. Finally, given that SSc is a disease associated with skin fibrosis, we also examined if these signatures may be relevant to SSc skin disease.

## 2 Materials and methods

### 2.1 Gene expression meta-analysis pipeline

**Figure 1:**
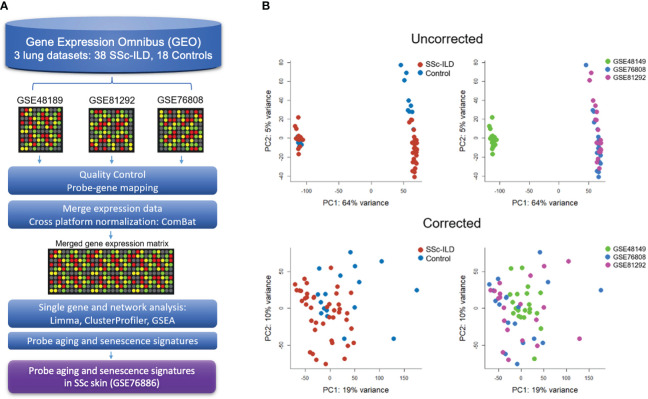
Gene expression meta-analysis pipeline **(A)** with PCA plots demonstrating successful merge after batch correction **(B)**.

A schematic overview of the meta-analysis pipeline is presented in **Figure 1A**. Publicly available microarray datasets from Gene Expression Omnibus (GEO) were searched for keywords “scleroderma”, “systemic sclerosis”, and “interstitial lung disease”. Samples were curated to only include lung tissue from adult patients meeting ACR 2019 criteria for SSc who carried a diagnosis of SSc-ILD. When a study included multiple tissue samples for a single subject, expression data from the lower lobe was favored given the predilection for disease in that area, and other samples were excluded to avoid overrepresentation of any individuals. Healthy controls from each respective study were also included. Clinical covariates including age, sex, and disease duration were not available for all individual samples and therefore not included in the study.

Raw data from each microarray dataset were downloaded from GEO. Processing first took place for each individual dataset according to their unique microarray platform, which included background correction, log2 transformation, normalization, and probe to gene mapping using R. LIMMA was utilized to process Illumina arrays, while SCAN.UPC from Bioconductor was utilized for Affymetrix arrays (20, 21). To be merged, expression data were mean-centered and reduced to genes common to all datasets. Cross-study normalization and dataset merging to create a single gene expression matrix were performed using ComBat, an empirical Bayes method, using individual study as a covariate (22). Principal component analysis (PCA) and boxplots were used to evaluate the successful batch correction and to detect outliers.

### 2.2 Differential gene expression

Differential gene expression analysis was performed using LIMMA (21). Genes considered differentially expressed (DEGs) between cases and controls were defined using a cutoff false discovery rate (FDR) *q*-value < 0.05 and fold change (FC) ≥ 1.2 or more. Unsupervised hierarchical clustering using the Ward algorithm was performed to generate heatmaps and identify gene expression clusters. Network analyses using Gene Ontology Enrichment Analysis and Gene Set Enrichment Analysis (GSEA) were conducted to identify genes and pathways significantly associated with SSc-ILD (p ¾0.05) (23, 24).

### 2.3 Cell type enrichment

Cell type enrichment analysis was performed using xCell v1.1, a gene-signature-based method, on the merged normalized data of SSc-ILD and controls (25). The existing compendium of cell type signatures were filtered to remove cell types not relevant to lung biology (i.e., osteoblasts, hepatocytes, keratinocytes, and neurons) leaving 45 cell types of the immune, stromal, and epithelial compartments. The false discovery rate was controlled by the Benjamini–Hochberg method, and the Wilcoxon test was used to determine differential cell enrichment between SSc-ILD cases and healthy controls. The significantly differentially enriched cell types, defined by threshold of adjusted *p <*0.1, were visualized with unsupervised hierarchical clustering using the Ward algorithm.

### 2.4 Probing aging and senescence signatures

Aging and senescence gene expression signatures have been established for the lung. Using bulk RNAseq of *in vitro* senescent cells, DePianto et al. defined an epithelial senescence (227 genes), fibroblast senescence (117 genes), and consensus senescence signature (11 genes) consisting of upregulated genes (17). Utilizing bulk RNAseq of healthy lungs across the lifespan, Lee et al. identified a lung-specific aging signature including 16 upregulated genes that were validated in publicly available data of healthy controls (8). These signatures were used to determine whether aging and cellular senescence were enriched in SSc-ILD. For senescence, each cell-type-specific signature was probed for overlapping genes within the merged gene expression matrix and visualized with unsupervised hierarchical clustering. The ratio of enrichment of each signature was determined using hypergeometric testing and GSEA. For aging, overlapping genes were examined individually, and gene expression between SSc and controls was compared using unpaired, two-sided t-test.

### 2.5 Telomere length measurement using TeloFISH

To further validate the findings of enhanced aging and cellular senescence in SSc-ILD, cell-specific telomere length was measured utilizing telomere quantitative fluorescence *in situ* hybridization assay (TeloFISH) in the lung tissue (26). Patient samples were obtained from a longitudinal, prospective cohort of patients with ILD at University of California San Francisco (UCSF) that was approved by the University Institutional Review Board at UCSF (IRB No. 10-00198). All participants provided written informed consent. Lung tissues from 15 age- and sex-matched subjects, including five SSc-ILD, five healthy controls, and five IPF, were fixed with 10% formalin overnight and embedded in paraffin and then sectioned to 4-μm thickness. Tissue slides were deparaffinized in xylene and rehydrated in ethanol gradient series. After heat-induced antigen retrieval, tissues were treated with 10 mg/ml RNase solution and incubated with 0.3 μg/ml peptide nucleic acid (PNA) TelC-Cy3 probe (Panagene, Korea) in 70% formamide buffer, heated to 78°C for 10 min then overnight at 20°C. Following washing and blocking, tissues were incubated with primary rabbit-anti SPC antibody, followed by secondary antibody. Tissues were mounted with DAPI. Images were acquired using a Zeiss Axio Imager 2 microscope, and telomere signal intensity was quantified in a blinded manner using MetaMorph imaging analysis software (Molecular Devices, Sunnyvale, CA). Pairwise comparisons of telomere length were made between groups using Kruskal–Wallis test.

### 2.6 Aging and senescence signatures in SSc skin

To examine if aging signatures were also relevant in the skin in SSc, we used a previously published gene expression dataset from the skin (GSE76886). Sample processing and sequencing has been described in detail previously (27, 28). PCA was used to visualize outliers. Metadata including SSc disease subtype (limited cutaneous vs. diffuse cutaneous) and age were utilized in downstream analysis. Gene expression data were probed for senescence signatures as described above. Specifically for aging, Lee et al. found a subset (10 genes) of the established aging genes upregulated in sun-exposed skin among publicly available data (8). These 10 genes were used for the aging analysis in the SSc skin.

## 3 Results

Three publicly available gene expression microarray datasets were identified (GSE48149, GSE81292, and GSE76808) (29–31). After curation and QC, removing four duplicate samples from GSE81292 and 31 non-SSc-ILD samples from GSE48149, a total of 38 SSc-ILD subjects and 18 healthy controls were included in the meta-analysis. Studies were successfully merged and batch corrected to overcome individual study differences (**Figure 1B**), and the merged gene expression matrix included 11,542 common genes for analysis.

### 3.1 Single gene and pathway analysis reveal markers of cellular senescence and shared features with IPF

**Figure 2:**
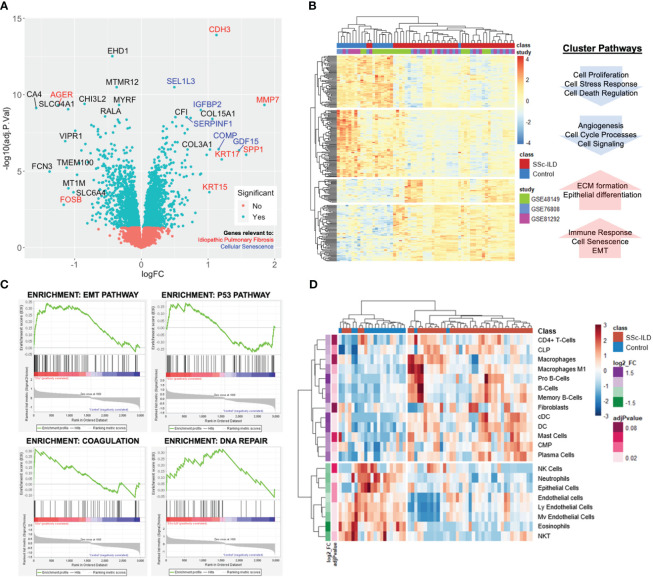
Lung gene expression meta-analysis of SSc-ILD and healthy controls. **(A)** Volcano plot of significant genes demonstrates overlap with IPF (red) and cellular senescence markers (blue). **(B)** Heatmap of significantly differentially expressed genes (FC>1.2, q<0.05) with each sample annotated by class (SSc-ILD vs. control) and individual study. Hierarchical clustering demonstrates clustering of cases and controls and reveals four distinct clusters with associated pathways. **(C)** Gene set enrichment analysis identifies EMT, p53, coagulation, and DNA repair pathways as most highly enriched in SSc-ILD. **(D)** Heatmap of cell enrichment calculated using xCell demonstrates clustering of cases and controls based on cell type and reveals increase in dendritic cells and loss of endothelial cells in SSc-ILD.

There were 586 differentially expressed genes (DEGs) (FC>1.2, q<0.05) between SSc-ILD and healthy controls with 264 genes upregulated and 322 downregulated in SSc-ILD (**Supplementary Table S1**). Among the top upregulated DEGs, notable markers included MMP7, a metalloproteinase involved in extracellular matrix (ECM) degradation and biomarker of pulmonary fibrosis; KRT17, an epithelial basal cell marker overexpressed in idiopathic pulmonary fibrosis; and SPP1, a biomarker of IPF. GDF15, a marker of cellular stress and senescence, and COMP were also found to be increased (**Figure 2A**). Hierarchical clustering of the top DEGs revealed separation between SSc-ILD and controls with the emergence of four distinct gene clusters. Pathway analysis of individual clusters demonstrates notable downregulation of cell processes, regulation of cell death, and angiogenesis, while upregulated pathways include ECM formation, epithelial differentiation, collagen organization, and senescence (**Figure 2B**; **Supplementary Table S2**). Gene set enrichment analysis revealed enrichment of eight hallmark pathways including notable enrichment of epithelial mesenchymal transition pathway (FDR q-value < 0.001), coagulation (FDR q-value = 0.02), P53 pathway (FDR q-value = 0.02), and DNA repair (FDR q-value = 0.05) (**Figure 2C**). Together, these analyses reveal upregulation of markers of cellular senescence and those described in IPF in SSc-ILD compared to controls.

### 3.2 Cell enrichment analysis reveals loss of endothelial cells

A total of 45 cell types were examined by cell type enrichment analysis including cells from the epithelial, stromal, and immune cell compartments. A total of 21 cell types were found to be significantly enriched in the lung tissue, including 13 enriched in SSc-ILD and eight enriched in controls (adj p-value < 0.1). Hierarchical clustering demonstrated distinct separation between cases and controls (**Figure 2D**). B cells, dendritic cells, and plasma cells were the most highly increased in SSc-ILD, while various types of endothelial cells, neutrophils, eosinophils, and epithelial cells were most significantly depleted in SSc-ILD compared to controls (adj p-value <0.05). Notably fibroblasts were only slightly increased in SSc-ILD.

### 3.3 Aging and senescence pathways are enriched in SSc-ILD

**Figure 3:**
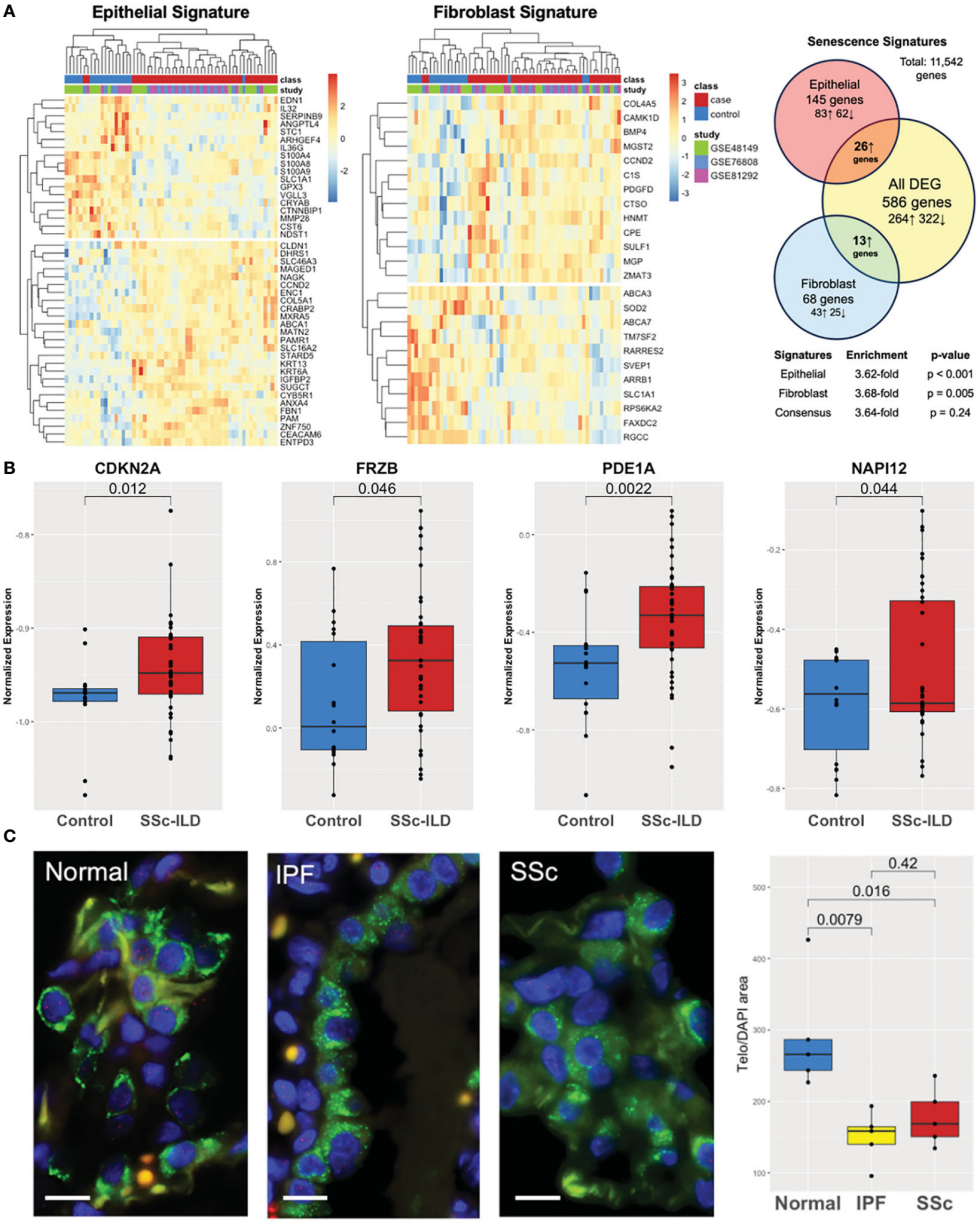
Aging and senescence are enriched in SSc-ILD. **(A)** Heatmap of lung epithelial and fibroblast senescence signatures annotated by class and individual study with hierarchical clustering demonstrating distinct patterns of expression in SSc-ILD compared to controls and 3.6- and 3.7-fold enrichment, respectively. **(B)** Aging genes are increased in SSc-ILD compared to controls. **(C)** TeloFISH demonstrates that telomere length is shortened in SSc-ILD vs. controls and comparable to IPF. Scale bar, 10 µm.

Both cell-specific senescence (DePianto et al.) and aging-specific (Lee et al.) genes were probed to examine if they were enriched in the SSc-ILD dataset. Among senescence signatures, 145 out of the 227 previously described epithelial genes and 68 out of the previously described 117 fibroblast genes were detected in our dataset. Hierarchical clustering of both epithelial and fibroblast senescence signatures demonstrated a distinct pattern of expression between SSc-ILD patients and healthy controls and found to have a 3.6- and 3.7-fold increase, respectively, in SSc-ILD compared to controls (**Figure 3A**). The signatures were also significant by GSEA (FDR ¾ 0.1, **Supplementary Figure S1**). When probing the aging gene signature, 8/16 genes upregulated in aging were present in the dataset with four significantly increased in SSc-ILD (*CDKN2A*, *FRZB*, *PDE1A*, and *NAPI12*) (**Figure 3B**). Overall, both aging- and cell-specific senescence signatures were enriched in SSc-ILD.

### 3.4 TeloFISH demonstrates shortened telomere length in SSc-ILD

To validate the aging-related findings in the publicly available data, we examined telomere length in the lung tissue in SSc-ILD. Given the shared markers and pathways between SSc-ILD and IPF and lack of understanding of the key pathological cell type driving SSc-ILD, we focused the telomere studies on type II alveolar cells, the stem cell of the lung epithelium implicated in IPF pathogenesis (32). Telomere staining was found to be significantly less intense in both SSc-ILD compared age matched control lungs (p = 0.016) and similarly significantly less intense in IPF (p = 0.008). Telomere intensity was similar between IPF and SSc-ILD (p = 0.42) (**Figure 3C**).

### 3.5 Aging and senescence signatures are enriched in the SSc skin

**Figure 4:**
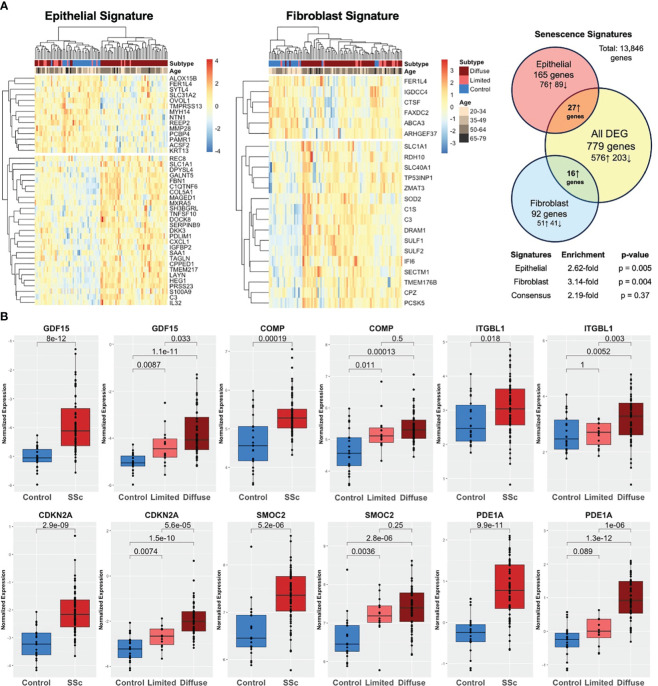
Aging and senescence are enriched in the SSc skin. **(A)** Heatmap of epithelial and fibroblast senescence signatures annotated by disease subtype (diffuse SSc vs. limited SSc vs. control) and age group. Hierarchical clustering demonstrating distinct patterns of expression in SSc skin compared to controls with 2.6- and 3.1-fold enrichment, respectively. **(B)** Aging genes are increased in the SSc skin compared to controls and expression associated with degree of skin severity (limited vs. diffuse cutaneous).

Both cell-specific senescence and aging-specific genes were probed to investigate their presence and relevance in SSc skin including 22 healthy controls and 70 SSc patients (GSE76886). Among the senescence signatures, 165 out of 227 epithelial genes and 92 out of 117 fibroblast genes were detected in the skin dataset. Hierarchical clustering of both epithelial and fibroblast senescence signatures demonstrated a distinct pattern of expression between SSc patients and healthy controls, with some separation by SSc cutaneous subtype (limited vs. diffuse cutaneous). Notably, the senescence signature expression did not cluster by age group (**Figure 4A**). Compared to all DEGs, the epithelial senescence signature and fibroblast senescence signature was found to have a 2.6- and 3.1-fold enrichment, respectively, in SSc skin disease compared to controls. Among aging-specific genes, 10 were reported to be increased in the aging sun-exposed skin in publicly available GTEx by Lee et al. Among these genes, six were present in our dataset, and four were significantly increased in SSc skin compared to controls (**Figure 4B**). Two of the most highly expressed senescence markers in SSc-ILD, namely, GDF15 and COMP, were also significantly increased in the SSc skin. Many of the aging and senescent genes demonstrated a stepwise increase between SSc subtypes with diffuse cutaneous having significantly increased expression of *GDF15*, *CDKN2A*, and *PDE1A*, suggesting that the degree of skin severity may be associated with aging and senescence gene expression. Overall, similar to the findings in SSc-ILD, aging- and cell-specific senescence signatures were enriched in SSc skin disease.

## 4 Discussion

Advanced age is a known risk factor for fibrotic lung disease and associated with worsened clinical outcomes and decreased survival (6). Cellular senescence, a hallmark of aging, is a state of replicative arrest, leading cells to adopt a senescence-associated secretory phenotype (SASP), which has been shown to have pro-fibrotic effects on the lung (10). Given their relevance to lung fibrosis pathogenesis, novel aging and senescence signatures have been established and validated in the lung. Our objective was to investigate the expression of the signatures in SSc-ILD to ascertain their presence and potential relevance to the disease. Through a gene expression meta-analysis of publicly available data, we demonstrate that markers of senescence (i.e., GDF15, COMP, and CDKN2A) and senescence-related pathways (i.e., P53 and EMT) are significantly upregulated in SSc-ILD compared to controls. We then took a hypothesis-driven approach and probed established aging- and cell-specific senescence signatures and found that both epithelial and fibroblast signatures and aging-related genes were significantly enriched in the lung tissue of SSc-ILD and in the SSc skin. Finally, the presence of telomere shortening, a hallmark of aging and senescence, in the lung epithelium of SSc-ILD further supports the possible mechanistic effects of accelerated aging in SSc-ILD pathogenesis.

Aberrant aging as a driver of pulmonary disease has been well established in several lung diseases including IPF and COPD; however, its role in SSc-ILD remains less clear (11, 33, 34). Previous studies have reported that the expression of p53 pathway, a key mediator in apoptosis and senescence, is increased in SSc-ILD as is the presence of p16 in the lung tissue of SSc-ILD (17, 19). Telomere length has also been found to be shortened in the peripheral blood of SSc-ILD patients, and the degree of shortening has been associated with clinical outcomes and survival (35). In fact, circulating antibodies to telomere-associated proteins have been identified in a subset of SSc patients, suggesting that autoimmunity may contribute to an aberrant aging phenotype (36). Our findings add to and strengthen the limited but growing body of literature that pathological aging and senescence may be implicated in SSc-ILD.

Several cell types and mechanisms have been proposed on how cellular senescence may promote the fibrosis of SSc-ILD. For example, endothelial cell senescence has been shown to inhibit angiogenesis and promoted endothelial to mesenchymal transition (EndoMT), which is thought to be profibrotic and can contribute to both dermal and pulmonary fibrosis (37, 38). In our dataset, we found that there was a significant loss of “normal” endothelial cells by cell enrichment analysis and that angiogenesis pathways were dysregulated in SSc-ILD compared to controls. Similarly, accumulation of senescent fibroblasts, which have increased ECM production and pro-inflammatory cytokine secretion, have been described in SSc, primarily in the skin, and murine models of pulmonary fibrosis, and may have a predilection for myofibroblast transformation (39, 40). Additional functional studies are needed to validate that aging mediators are drivers of fibrosis in SSc-ILD and, importantly, better define whether the fibrosis propagated in the context of aging and cellular senescence stems from the epithelial, stromal, and/or immune compartments.

The role of senescent cells in the development of pulmonary fibrosis is well described in IPF, and due to the mechanistic and therapeutic implications, there is interest in understanding shared pathology between interstitial lung diseases (10, 17). SSc-ILD has known shared features with IPF including later age of onset, male predilection, and a subset of SSc-ILD patients having identical histopathological findings of usual interstitial pneumonia (UIP) pattern of fibrosis. In our analysis, we found significant overlapping features between SSc-ILD and IPF, suggesting that there may be in part a shared pathobiology between the two diseases. On a single-gene level, MMP7, a matrix metalloproteinase responsible for ECM breakdown, was the top differentially expressed gene in SSc-ILD. MMP7 is a well-described biomarker in IPF that is associated with clinical outcomes and thought to promote epithelial to mesenchymal transition and profibrotic mediators in IPF (41). In SSc-ILD, MMP7 has been shown to be expressed in alveolar type II cells and bronchial epithelium in a similar fashion to IPF lung tissue (42). Similarly, SPP1, KRT17, and CDH3, which are upregulated in SSc-ILD, have all been described to be dysregulated in IPF (43–46). Telomere dysfunction and shortened telomeres are a hallmark of IPF pathogenesis (11, 14, 15). We demonstrate telomeres are significantly shortened in SSc-ILD in type II alveolar cells, the pathological cell type of IPF, compared to controls. ATII cells, which are responsible for maintaining pulmonary homeostasis, when senescent, have been shown to be among the key drivers of the initial fibrosis in IPF. While the roles of ATII cells and other epithelial cells in SSc-ILD need to be elucidated, our data demonstrates that telomere attrition is present at the tissue level in SSc-ILD to a similar extent as IPF.

The rarity of SSc and difficulty of obtaining affected tissue, especially lung tissue, has limited the sample size of translational studies in the field. By leveraging the publicly available data and utilizing gene expression meta-analysis approach, we were able to increase sample size and patient diversity in a rare disease allowing for more robust findings compared to individual studies. However, there are limitations to our study. Demographic and clinical characteristic data such as age, sex, medication use, and disease duration were not available on an individual case basis for lung datasets and therefore not utilized for this study. Each dataset did cite using age-similar individuals between cases and controls in their original studies; however, future work can provide further clarity on the impact of these variables. This meta-analysis was cross-sectional in nature, providing a snapshot of gene expression in an otherwise dynamic disease. Another limitation is that this study utilized gene expression available from microarray data, given no bulk RNAseq studies present of SSc lung tissue, while the aging and senescence signatures were developed using bulk RNAseq. There were therefore several genes described by these signatures that were not detected in our dataset once merged.

In conclusion, our study contributes to the growing body of evidence that aberrant aging may play a role in SSc-ILD pathogenesis. We demonstrated that aging and senescence signatures are upregulated in SSc-ILD and that telomere dysfunction is present in the lung tissue of SSc-ILD. These findings have both mechanistic and therapeutic implications; however, further studies need to be done. Future work includes examining these signatures on a single-cell level to determine the cell-type-specific transcriptional profiles of senescent cells of interest in SSc-ILD and functional studies to understand their implications on disease pathogenesis.

## Data availability statement

The original contributions presented in the study are included in the article/**Supplementary Material**. Further inquiries can be directed to the corresponding author.

## Ethics statement

The studies involving humans were approved by Institutional Review Board (IRB) STU00004428. The studies were conducted in accordance with the local legislation and institutional requirements. All participants provided written informed consent.

## Author contributions

MY: Conceptualization, Data curation, Investigation, Methodology, Writing – original draft, Writing – review & editing. SL: Formal analysis, Investigation, Writing – review & editing. JN: Investigation, Methodology, Supervision, Writing – review & editing. MH: Writing – review & editing, Investigation. PW: Conceptualization, Formal analysis, Investigation, Methodology, Resources, Supervision, Writing – review & editing. MS: Conceptualization, Formal analysis, Investigation, Methodology, Resources, Supervision, Writing – review & editing.
