# Supplementary figures and images for: Gene expression meta-analysis reveals aging and cellular senescence signatures in scleroderma-associated interstitial lung disease
Source: Front Immunol. 2024 Jan 25;15:1326922. doi: 10.3389/fimmu.2024.1326922 (PMC10859856; doi:10.3389/fimmu.2024.1326922)

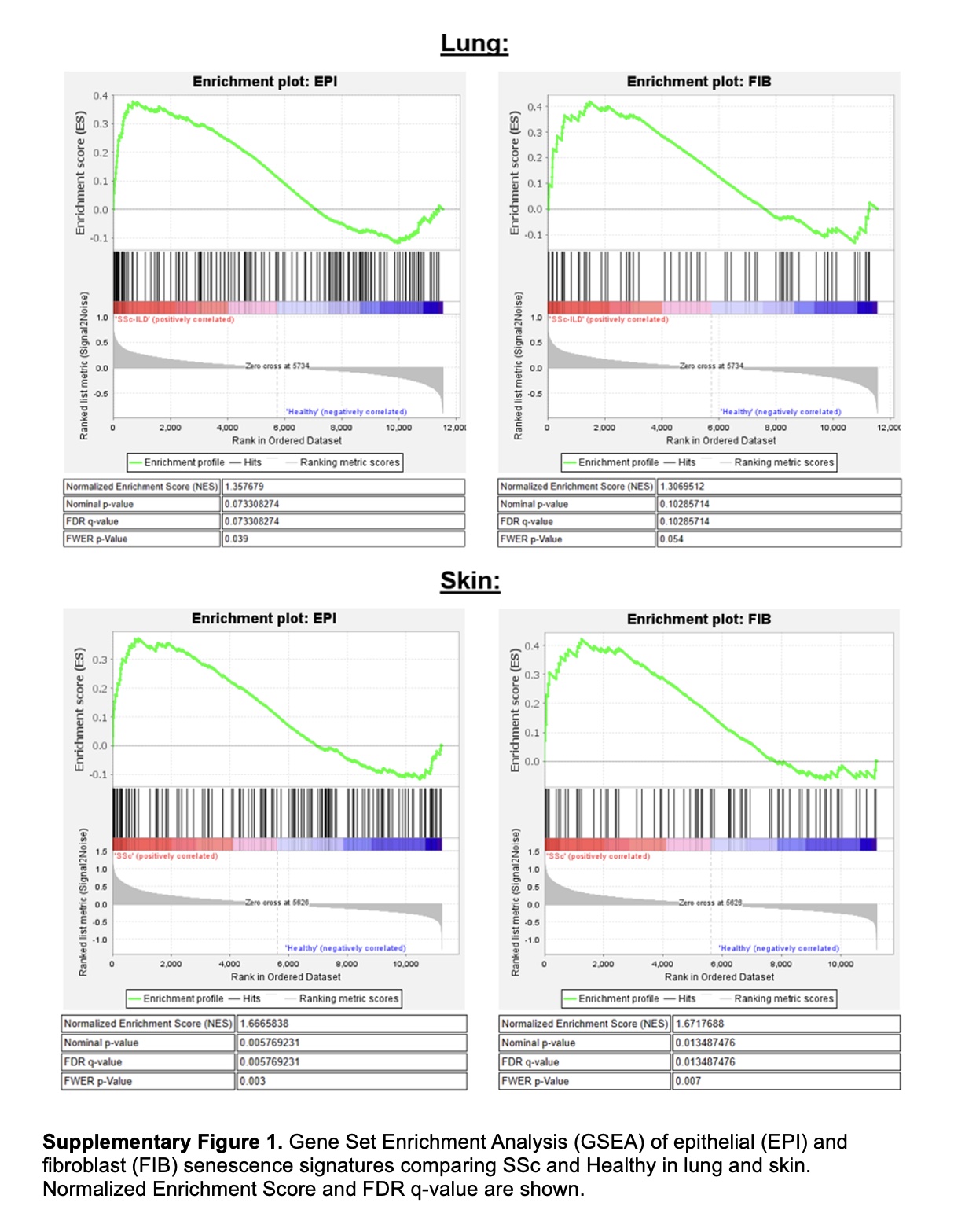

Supplement: Supplementary file 1 [file Image_1.jpeg]
